# Cervical Screening Results Leading to Detection of Adenocarcinoma in Situ of the Uterine Cervix

**DOI:** 10.31557/APJCP.2019.20.2.377

**Published:** 2019

**Authors:** Santipap Srisomboon, Charuwan Tantipalakorn, Kittipat Charoenkwan, Jatupol Srisomboon

**Affiliations:** *Department of Obstetrics and Gynecology, Faculty of Medicine, Chiang Mai University, Chiang aMai, Thailand. *

**Keywords:** Adenocarcinoma in situ -uterine cervix-cervical screening results

## Abstract

**Background:**

Adenocarcinoma in situ (AIS) of the uterine cervix is a preinvasive lesion of the invasive adenocarcinoma. We analyzed the cervical screening results leading to detecting the AIS lesions including the co-existence of AIS lesions with high-grade squamous intra-epithelial lesions (HSIL) and invasive carcinoma.

**Methods:**

Women who were diagnosed and received treatment for AIS at Chiang Mai University Hospital between January 1, 2007 and August 31, 2016 were retrospectively reviewed. The inclusion criteria were the women who had pathological diagnosis of AIS obtained from cervical punch biopsy or excisional cone biopsy with either loop electrosurgical excision procedure (LEEP) or cold-knife conization (CKC). The patient characteristics, diagnostic work-up and treatment details were reviewed, including the cervical screening results prior to the diagnosis of cervical AIS, pathologic results of excisional cone biopsy and hysterectomy specimens.

**Results:**

During the study period, 75 women with AIS pathology undergoing excisional cone biopsy with either LEEP (n=62) or CKC (n=13) were identified. The abnormal cytologic screening leading to detection of AIS was the squamous cell abnormality accounting for 57.3%. Abnormal glandular cytology accounted for 37.3%. The most common abnormal cervical screening results was HSIL cytology (n = 25) followed by AIS cytology (n = 13). Normal cytology was noted in 4 women in whom 3 were positive for HPV 18 and 1 had AIS on the endocervical polyp. AIS coexisted with HSIL and invasive carcinoma were detected in cone biopsy specimens in 21 (28%) and 29 (38.7%) patients, respectively.

**Conclusion:**

The majority of cervical screening results leading to detection of cervical AIS was the squamous cell abnormality accounting for 57.3% in which, HSIL cytology was the most common. Abnormal glandular cytology accounted for only 37.3%. Diagnostic cone excision is recommended if AIS lesion is noted in cervical biopsy specimen since nearly 40% have coexisting invasive lesions.

## Introduction

Cervical cancer is the second most common cancer in females worldwide and the most common in low and middleincome countries (Ferlay et al., 2015). In general, squamous cell carcinoma and adenocarcinoma are the 2 major histological types of cervical cancer. Adenocarcinoma, accounting for 10 20% of all cervical cancers, has been increasing in the last two decades, unlike those of squamous cell carcinoma which is proportionally decreasing (Gustafsson et al., 1997; Vizcaino et al., 2000). Adenocarcinoma in situ (AIS) of the uterine cervix has been recognized as a pre-invasive or precursor lesion of invasive adenocarcinoma due to its morphological futures, which are similar to those of invasive adenocarcinomas. AIS lesion frequently coexists with invasive adenocarcinoma on excisional specimens and has similar human papillomavirus (HPV) types as have been detected in invasive adenocarcinoma (Zaino, 2002). 

AIS lesion of the uterine cervix is almost always asymptomatic and is generally not visible upon gross examination. It is typically detected after a diagnostic work-up for abnormal cervical cytology screening. The average age of patients with AIS is approximately 5 years younger than those with early invasion, supporting the potential for cytologic screening to prevent this disease. Most cases of invasive adenocarcinoma develop from an AIS precursor located within or just proximal to the transformation zone. There is a window of approximately 5 years between clinically detectable AIS and early invasive adenocarcinoma, indicative of an opportunity for screening and detection before progressing to invasion (Lee and Flynn, 2000). The ability to detect its precursor, AIS, would certainly decrease the incidence of invasive adenocarcinoma. However, limitations of conventional methods in detecting glandular lesions exist. Pap cytology had a sensitivity of only 45 %in detecting glandular diseases (Krane et al., 2001). Since Pap cytology commonly fails to detect the AIS lesions, accordingly, this retrospective study was conducted to determine the screening results leading to the detection of cervical AIS, in addition, the co-existence of high-grade squamous intra-epithelial lesions (HSIL) and invasive carcinoma was also evaluated.

## Materials and Methods

After approval of the Research Ethics Committee of Chiang Mai University Hospital, women who were diagnosed and received treatment for adenocarcinoma in situ (AIS) of the uterine cervix between January 1, 2007 and August 31, 2016 were retrospectively reviewed. The data resources for review were medical records in the colposcopy clinic of Obstetrics and Gynecology Department and the computerized database of Chiang Mai University Hospital. The patient characteristics, diagnostic work-up and treatment details were reviewed, including the age ,parity, contraception methods ,HIV status, number of sexual partners ,smoking history, cervical screening results prior to the diagnosis of cervical AIS, pathologic results of cervical punch biopsy, cone biopsy and hysterectomy specimens. The 2001 Bethesda System for nomenclature of cervical cytology was used to report the cytology results (Solomon et al., 2002). The inclusion criteria were the women who had pathological diagnosis of AIS obtained from cervical punch biopsy or diagnostic excision procedure with either loop electrosurgical excision procedure (LEEP) or cold-knife conization (CKC). Women who were pregnant or had history of gynecologic cancer were excluded. 

**Table 1:** Preceding Cervical Cytology, Pathology, Cone Margin Status Stratified by Type of Excisional Cone Biopsy^a^

Characteristics	All women	LEEP	CKC
	(n = 75)	(n = 62)	(n = 13)
Preceding cytology			
NILM	4 (5.33)	4 (6.45)	0
Squamous cell bnormality	43 (57.33)	39 (62.90)	4 (30.77)
ASC-US	4 (5.33)	4 (6.45)	0
ASC-H	7 (9.33)	6 (9.68)	1 (7.69)
LSIL	4 (5.33)	3 (4.84)	1 (7.69)
HSIL	25 (33.33)	23 (37.10)	2 (15.38)
Squamous cell carcinoma	3 (4.00)	3 (4.84)	0
Glandular cell abnormality	28 (37.33)	19 (30.65)	9 (69.23)
AGC^b^	7 (9.33)	7 (11.29)	0
AIS	13 (17.33)	6 (9.68)	7 (53.85)
Adenocarcinoma	8 (10.67)	6 (9.68)	2 (15.38)
Pathology			
Pure AIS	25 (33.33)	20 (32.26)	5 (38.46)
Mixed AIS with HSIL	21 (28.00)	20 (32.26)	1 (7.69)
AIS coexisted with invasive carcinoma	29 (38.67)	22 (35.48)	7 (53.85)
Squamous cell carcinoma	4 (5.33)	4 (6.45)	0
Adenocarcinoma	25 (33.33)	18 (29.03)	7 (53.85)
Cone margin status			
Negative	24 (32.00)	19 (30.65)	5 (38.46)
Positive	51 (68.00)	43 (69.35)	8 (61.54)

LEEP, loop electrosurgical excision procedure; CKC, cold-knife conization; NILM, negative for intraepithelial lesion and malignancy; ASC-US, atypical squamous cells of undetermined significance; ASC-H, atypical squamous cells cannot exclude HSIL; LSIL, low-grade squamous intraepithelial lesion; HSIL, high-grade squamous intraepithelial lesion; AGC, atypical glandular cells; AIS, adenocarcinoma in situ; ^a^,Values are expressed as number (percentage)

bIncluded AGC-not otherwise specified (1 case); AGC-favor neoplasia (2 cases) and atypical glandular cells only (3 cases)

**Table 2:** Residual Lesions Stratified by Type of Excisional Cone Biopsy, Cone Margin Status and Endocervical Curettage Results in 61 Women Undergoing Subsequent Hysterectomy

Variables	Number	Residual lesions		P value
		Absence (%)	Presence (%)	
Type of excision				
LEEP margins	48			
Positive	37	11 (29.73)	26 (70.27)	0.004
Negative	11	9 (81.82)	2 (18.18)	
CKC margins	13			
Positive	8	4 (50.00)	4 (50.00)	0.6
Negative	5	4 (80.00)	1 (20.00)	
ECC results				
Normal	28	17 (60.71)	11 (39.29)	
Abnormal	13	4 (30.77)	9 (69.23)	0.08
Not done	20	7 (35.00)	13 (65.00)	

LEEP, loop electrosurgical excision procedure; CKC, cold-knife conization; ECC, endocervical curettage

**Figure 1:**
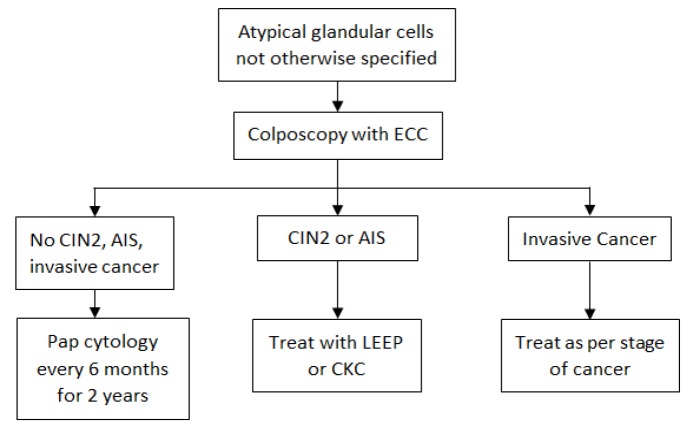
Management of Women with Atypical Glandular Cells not Otherwise Specified. CIN2, cervical intraepithelial neoplasia grade 2; LEEP, loop electrosurgical excision procedure; CKC, cold-knife conization; AIS, adenocarcinoma in situ; ECC, endocervical curettage

**Figure 2:**
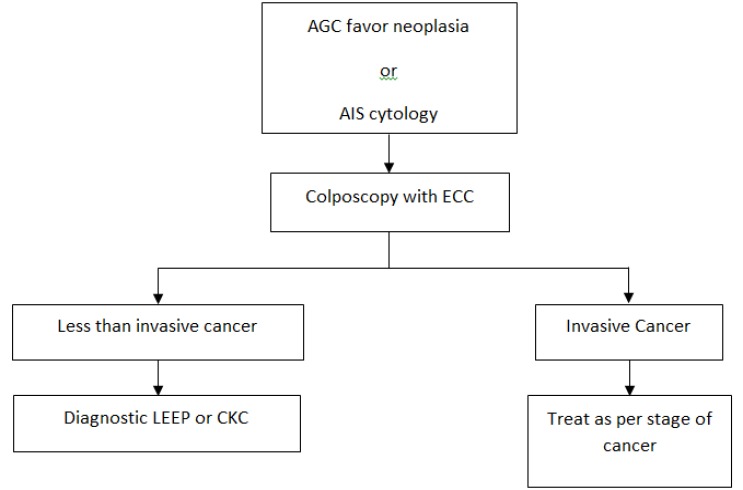
Management of Women with Atypical Glandular Cells Favor Neoplasia and AIS. AGC, atypical glandular cells; LEEP, loop electrosurgical excision procedure; CKC, cold-knife conization; AIS, adenocarcinoma in situ; ECC, endocervical curettage

Management of women with glandular cell abnormality on cytology and AIS on cytopathology are showed in Figure 1 and 2. In women with histological AIS on cervical punch biopsy, a diagnostic excisional cone procedure with endocervical curettage (ECC) would be carried out to exclude invasive carcinoma in the adjacent areas regardless of prior screening results. Women with histological AIS on cone specimens who had completed childbearing were treated with total hysterectomy. If future fertility was desired, close follow-up with Pap cytology and colposcopy was recommended to the patients. In patients whose margins of the cone specimens were positive or any lesions were present in the ECC specimens, re-excision or hysterectomy would be carried out depending on pathology of the cone specimens, the result of ECC and patient’s preference of fertility preservation. If invasive carcinoma was detected in cone specimens, the patients were treated as per stage of the disease. 

The statistical analysis was performed using SPSS version 21.0 (IBM Corp. Released 2012; IBM SPSS Statistics for Windows, Version 21.0. Armonk, NY: USA). The descriptive data were presented as percentage / range or means + SD, as appropriate. Mann-Whitney U test, Student’ t test, chi-square test, and Fisher exact test were used for statistical analysis when appropriate. P value of less than 0.05 was considered statistically significant. All statistical tests were of two-sided significance.

## Results

During the study period, 75 women with AIS pathology who underwent excisional cone biopsy with either LEEP or CKC were identified. The mean age of the women was 45.9 years with a range of 26-66 years. Eight (10.7%) women were nulliparous. Anti-HIV testing was negative in all patients. No contraception was used in 23 (30.7%) women, while 27 (36%) used oral contraceptive pills, 7 (9.3%) used injectable contraception, 16 (21.3%) had tubal sterilization and 2 (2.7%) used condom contraception. Nineteen (25.3%) women were postmenopausal.

Table 1 shows preceding cervical cytology in women with AIS pathology in cervical punch biopsy, excisional cone biopsy specimens and cone margin status stratified by type of cone biopsy. Among the 75 cytology results, 66 were obtained from conventional Pap smears and 9 from ThinPrep cytology accompanied with HPV co-testing. The most common abnormal cervical screening results was HSIL cytology (n = 25) followed by AIS cytology (n = 13). Although AIS is the disease of glandular epithelium, the preceding abnormal cytologic screening in our study was the squamous cell abnormality accounting for 57.3%. Abnormal glandular cytology accounted for 37.3%. Among 13 women with AIS cytology, 7 and 6 had AIS and invasive adenocarcinoma in cone biopsy specimens, respectively. The final pathology of 8 women with adenocarcinoma on cytology was pure AIS (2), mixed AIS and HSIL (3) and invasive adenocarcinoma (3).

HPV testing was performed in conjunction with cervical cytology in 9 women and 8 was positive (6 for HPV 18 and 2 for unknown high-risk HPV type). Among 4 women with normal cytology, 3 were positive for HPV 18 and 1 had AIS on the endocervical polyp. Positive HPV 18 was found in 2 women with AGC cytology and 1 with adenocarcinoma on cytology. HPV testing was positive for unknown type in 1 women with ASC-US cytology and another one with HSIL cytology. AIS coexisted with HSIL and invasive carcinoma were detected in cone biopsy specimens in 21 (28%) and 29 (38.7%) patients, respectively. The resection margins of the cone biopsy specimens were positive in 51 (68%) patients and were not significantly different when compared between LEEP specimens and CKC specimens (p = 0.74)

Table 2 shows the residual lesions stratified by type of the excisional cone biopsy, cone margin status and endocervical curettage (ECC) results in 61 women who underwent subsequent hysterectomy. Among 48 women undergoing prior LEEP, 28 (58.3%) had residual lesions which was higher than those of 5 (38.4%) in 13 women with prior CKC. However, the difference was not statistically significant (p = 0.23). In the LEEP group, the prevalence of residual diseases was significantly associated with the resection margin status (p = 0.004). In comparison with the CKC group, the prevalence of residual diseases was not significantly related to the cone margin status. However, the number of patients in the CKC group was too small. ECC was performed in 41 women after excisional cone biopsy. Among 20 women in whom ECC was not carried out, the preceding cervical cytology results were normal (3), ASC-US (4), LSIL (4) ASC-H (2) and HSIL (7). The prevalence of residual diseases in subsequent hysterectomy specimens appeared to be higher in women with abnormal ECC results (69.2%) when compared with that of normal ECC results (39.3%), but the difference was not significant (p = 0.08).

## Discussion

Our study showed that among 75 women with a diagnosis of AIS of the uterine cervix, the most common preceding cervical cytology screening leading to the detection of this glandular disease was the squamous cell abnormality accounting for 57.3 %. The glandular cell abnormality including the AIS cytology accounted for only 33.3%. These findings were different from the previous retrospective studies reporting the glandular cell abnormality on cervical cytology prior to the detection of AIS ranging from 50 to 69 % and squamous cell abnormality ranging from 26 to 31 % (Shin et al., 2002; Mitchell et al., 2004). Among squamous cytologic abnormality, the most common were HSIL cytology ranging from 76 to 79 % (Geier et al., 2001; Schnatz et al., 2006). HSIL cytology (33.3%) was also the most common abnormal screening results in our study followed by the AIS cytology accounting for 17.3%. The lesions of AIS preferentially extend into the endocervical canal and occur in the deeper portions of the endocervical clefts beneath the transformation zone rather than at the surface as with the squamous lesions. Consequently, cytological screening frequently fails to detect the glandular lesions. Accordingly, Pap cytology in patients with AIS may be normal or squamous cell abnormality depending on the associated squamous lesions.

Of interest, normal cytologic screening was noted in 4 (5.3%) patients with AIS lesions in our study. Of these 4 patients, 3 had positive HPV testing for HPV 18, the remaining 1 had AIS on the endocervical polyp. Normal cervical cytology was found approximately 4 % in women with pathological diagnosis of AIS (Geier et al., 2001; Schnatz et al., 2006). AIS lesions have similar type of HPV as have been detected in invasive adenocarcinoma of the cervix (Zaino, 2002). The most common HPV types found in AIS lesion are HPV 16 and 18 accounting for more than 90% (Schnatz et al., 2006; Rabelo-Santos et al., 2009; Quint et al., 2010; Ault et al., 2011). In a meta-analysis of HPV prevalence in glandular lesions of the cervix, the HPV 16 and HPV 18 positivity rates in AIS lesion were 49% and 52%, respectively (Guan et al., 2013). 

It was shown that HPV type specific risks for AIS and adenocarcinoma are not the same as for squamous cell carcinoma, with HPV18 being consistently observed to be pref-erentially associated with AIS (Dahlstrom et al., 2010; Andersson et al., 2013). HPV 18 is more frequently detected in adenocarcinoma than in squamous cell carcinoma (Clifford and Franceschi, 2008). Currently, it is well recognized that HPV testing is more sensitive than cytology in cervical cancer screening especially AIS and adenocarcinoma (Wright et al., 2004; Meijer et al., 2009; Katki et al., 2011). Concurrent HPV testing resulted in early detection of adenocarcinoma, 17 of 27 (63%) women with adenocarcinoma were found to have HPV-positive but cytology-negative screening results (Katki et al., 2011). Pap smear had a sensitivity of only 43% in detecting AIS lesions(Kietpeerakool et al., 2006). In 2 clinical trials of quadrivalent HPV vaccine, HPV testing combined with cervical cytology was used to evaluate the efficacy of HPV vaccine. AIS lesions occurred in 22 women in whom 21(95.5%) had positive HPV testing while only 2 had abnormal glandular cytology. Furthermore, colposcopy failed to detect AIS in all cases (Ault et al., 2011). Colposcopic appearance is not specific to identify the lesions of AIS. Colposcopic evaluation and sampling are more difficult to detect AIS lesion because of the location of lesions within the endocervical canal or beneath the transformation zone. The findings of low sensitivity of cytology in screening glandular diseases strongly support the addition of HPV testing to cytology to improve screening performance. We suggest to keep in mind that glandular diseases may be present when manage HPV 18-positive women regardless of cytology results.

AIS coexisted with HSIL and invasive carcinoma, in our study were found in 21 (28%) and 29 (38.7%) patients, respectively. In the meta-analysis of 33 studies, approximately 55% of women with AIS were found to have a coexisting squamous neoplasia (Salani et al., 2009). The co-existence of both glandular and squamous neoplasia reflects the similar etiology between the 2 types of cervical cancer precursors. Because of the location of the AIS inside the endocervical canal and in the glandular clefts, the Pap cytology of both squamous and glandular lesions may indicate only squamous cell abnormality. In the study by Andersson et al., (2013) AIS was detected in 64.3% of the patients due to accompanying squamous cell abnormalities, but in only 27% due to glandular cell abnormalities. This may be due to the relatively low sensitivity of cytology screening which is ineffective in reducing the incidence of adenocarcinoma. When glandular and squamous neoplasia coexist, the squamous component is more likely to be noted colposcopically because of its prominent features, especially when the squamous lesions are extensive or high grade. Glandular lesions may be adjacent to the squamous lesion, be sandwiched between 2 squamous lesions, lie under or above the squamous lesion. Thus, the squamous lesion is frequently diagnosed from colposcopy-guided biopsy, with AIS being detected incidentally in a subsequent excisional cone biopsy or in a hysterectomy specimen. Invasive carcinoma, if detected concurrently, should be further evaluated and managed as appropriate.

When AIS is found on punch biopsy or when AIS is suspected cytologically or colposcopically but not proven histologically, a diagnostic excisional cone biopsy must be performed. The purpose is to confirm the diagnosis, assess the extent of lesion and exclude the coexisting invasive carcinoma. In our study, we preferred LEEP rather than CKC. We noted that overall , the cone margins were positive in 68% of the patients and were not significantly different when compared between LEEP specimens and CKC specimens. In the systematic review of 35 studies on excision procedures for AIS, it was shown that LEEP had a clinically significantly higher rate of positive cone margins when compared with CKC (51% vs. 30%) (Baalbergen and Helmerhorst, 2014). In general gynecological practice, CKC is favored over LEEP for women with AIS due to its greater depth and larger volume of specimens, more easily interpretable resection margins and lower rate of positive margins (Kietpeerakool et al., 2012; Costales et al., 2013). However, the Updated Consensus Guidelines of the American Society for Colposcopy and Cervical Pathology (ASCCP) allowed any diagnostic excision method for AIS lesion with carefully keeping specimen intact, margin interpretable, avoiding fragmentation of the specimens including ‘‘top-hat’’ serial endocervical excisions. This may require the use of larger loops than those employed to excise visible squamous lesions (Massad et al., 2013). 

The risk of residual diseases after excisional cone biopsy in our study was significantly associated with the resection margin status in patients undergoing LEEP. The risk of residual diseases in the CKC group seemed to be higher in patients with involved cone margins but not statistically significantly when compared with those who had negative margins. However, the number of patients (13) in this group was too small to conclude. Systematic review of 35 studies on excision procedures for AIS showed that the risk of residual disease was significantly associated with cone margin status, i.e. 16.5% vs. 49% in negative and positive cone margins, respectively. In addition, the risks of residual invasive carcinoma in subsequent surgical specimens after a negative and positive cone margins were 0.6% and 5.9%, respectively (Baalbergen and Helmerhorst, 2014). The risk of residual lesions in patient with AIS is quite high despite negative cone margins because the pattern of this glandular neoplasia is often multifocal and located beyond the proximal end of the endocervical cutting edge of the cone. Accordingly, it is recommended that women with AIS who have completed childbearing should undergo hysterectomy to reduce the risk of adenocarcinoma.

Among 61 women undergoing subsequent hysterectomy in our study,33 (54%) had residual diseases in hysterectomy specimens. The risk of residual lesions appeared to be higher in women with abnormal ECC results (69.2%) comparing with that of normal ECC results (39.3%), however, the difference was not significant. Of note, nearly 40% of patients had residual disease despite normal ECC results. Performing endocervical sampling is generally recommended after colposcopy and excisional cone biopsy in managing women with glandular cell abnormality on cytopathology, although the benefit of such procedure is conflicting in prediction of residual disease (Denehy et al., 1997; Lea et al., 2002). ECC result is considered as a part of evaluating margin status, i.e. abnormal ECC is a positive margin. Our findings support that negative ECC result after excisional cone biopsy does not guarantee the absence of disease in the residual cervix. Because the nature of AIS lesions are frequently located deep in endocervical clefts and invaginated within the cervical stroma, the lesions might not be scraped during ECC. As a result, normal ECC result does not necessarily ensure that the lesion has been completely excised. 

The strength of the present study was the inclusion of patients who were treated at a single institution with same management protocol and all the cytopathologic specimens were examined by expert gynecologic pathologists. However, the limitation of the study was the retrospective by nature, some data were not available, e.g. patient and physician preferences that might affect the selection of cone biopsy methods (LEEP vs. CKC). Due to the small number of patients with this rare disease, the clinical significance of cone margin status in women undergoing CKC could not be concluded. In addition, the treatment outcomes especially recurrence after definitive treatment for AIS were not available.

In conclusion, the majority of cervical screening results leading to detection of AIS, the pre-invasive glandular lesion of invasive adenocarcinoma of the uterine cervix was abnormality of squamous cells accounting for 57 % in which, HSIL cytology was the most common. Abnormal glandular cytology accounted for only 37 %. Diagnostic cone excision is recommended if AIS lesion is noted in cervical biopsy specimen since nearly 40% have coexisting invasive lesions.

## Conflict of Interest

The authors have no conflict of interest to disclose.
